# Supplementary figures and images for: Mesenchymal stem cell suppresses the efficacy of CAR-T toward killing lymphoma cells by modulating the microenvironment through stanniocalcin-1
Source: eLife. 2023 Feb 13;12:e82934. doi: 10.7554/eLife.82934 (PMC10019890; doi:10.7554/eLife.82934)

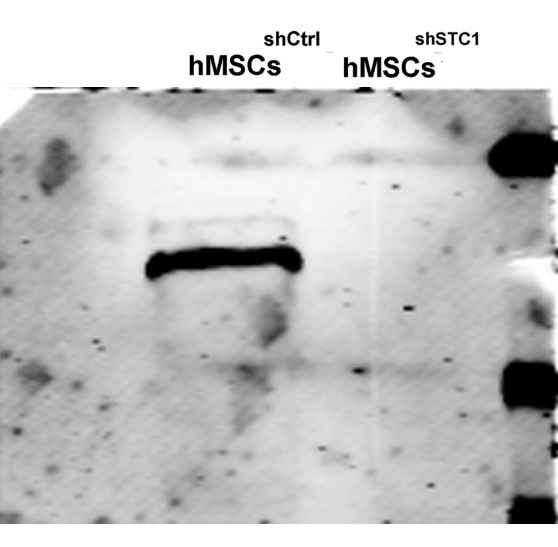

Supplement: Figure 1—source data 1. [file elife-82934-fig1-data1.zip › Fig 1A STC1(repeat) with labeling.tif]

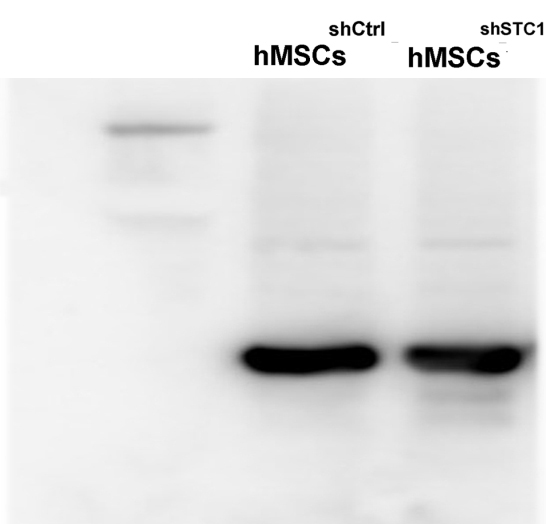

Supplement: Figure 1—source data 1. [file elife-82934-fig1-data1.zip › Fig1A GAPDH with labeling.jpg]

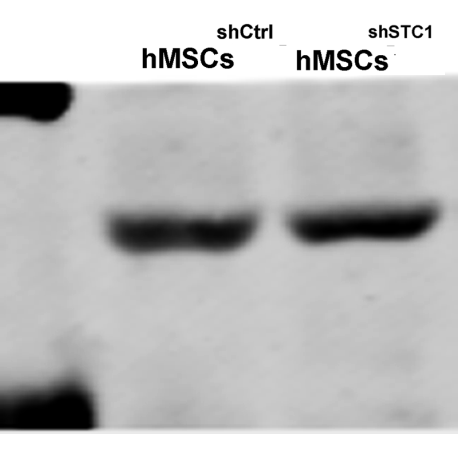

Supplement: Figure 1—source data 1. [file elife-82934-fig1-data1.zip › Fig1A GAPDH repeat with labeling.tif]

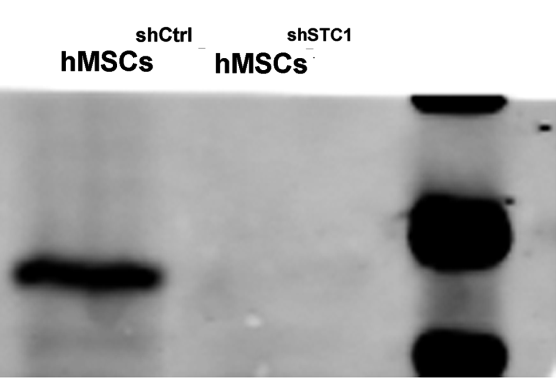

Supplement: Figure 1—source data 1. [file elife-82934-fig1-data1.zip › Fig1A STC1 with labeling.tif]

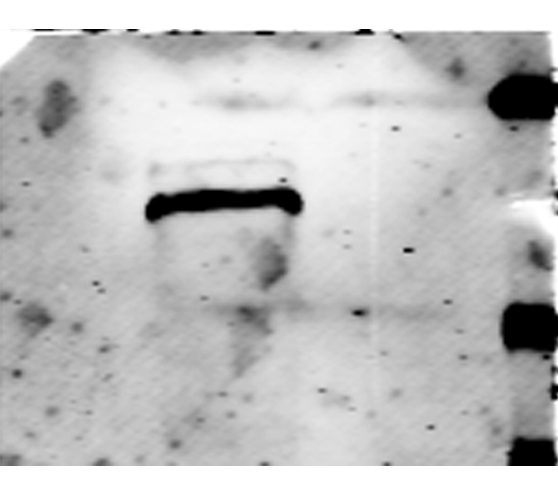

Supplement: Figure 1—source data 2. [file elife-82934-fig1-data2.zip › Fig 1A STC1(repeat).tif]

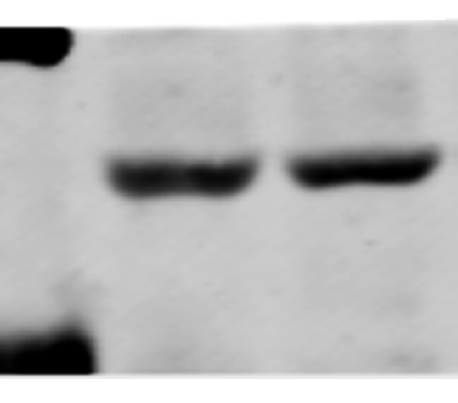

Supplement: Figure 1—source data 2. [file elife-82934-fig1-data2.zip › Fig1A GAPDH repeat.tif]

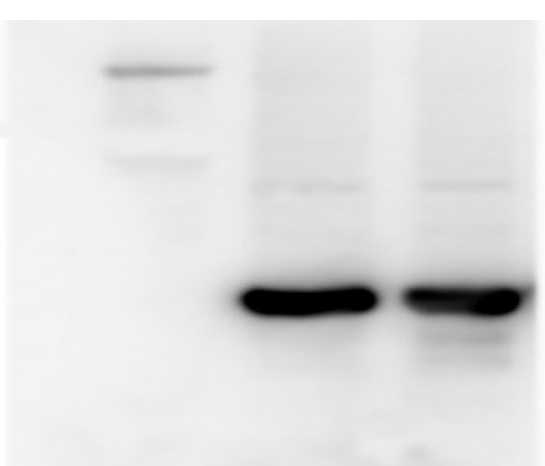

Supplement: Figure 1—source data 2. [file elife-82934-fig1-data2.zip › Fig1A GAPDH.jpg]

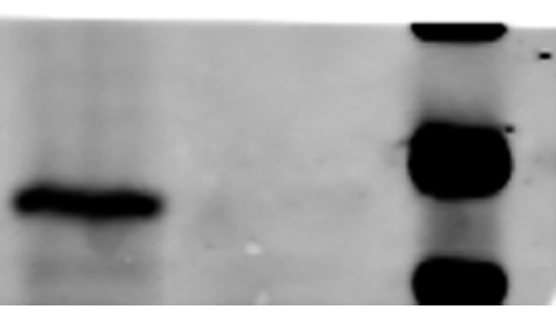

Supplement: Figure 1—source data 2. [file elife-82934-fig1-data2.zip › Fig1A STC1.tif]

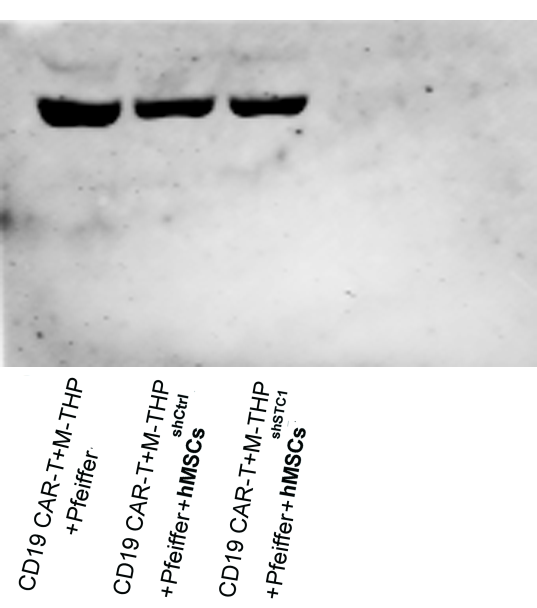

Supplement: Figure 2—source data 4. [file elife-82934-fig2-data4.zip › Fig2F GAPDH repeat.tif]

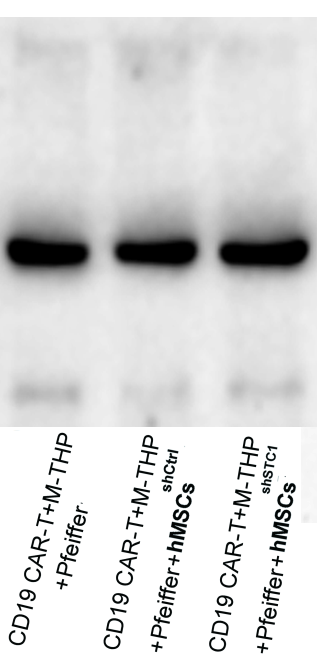

Supplement: Figure 2—source data 4. [file elife-82934-fig2-data4.zip › Fig2F GAPDH.tif]

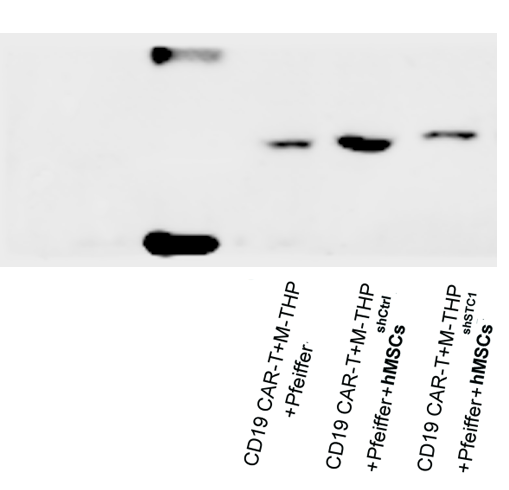

Supplement: Figure 2—source data 4. [file elife-82934-fig2-data4.zip › Fig2F IDO repeat.tif]

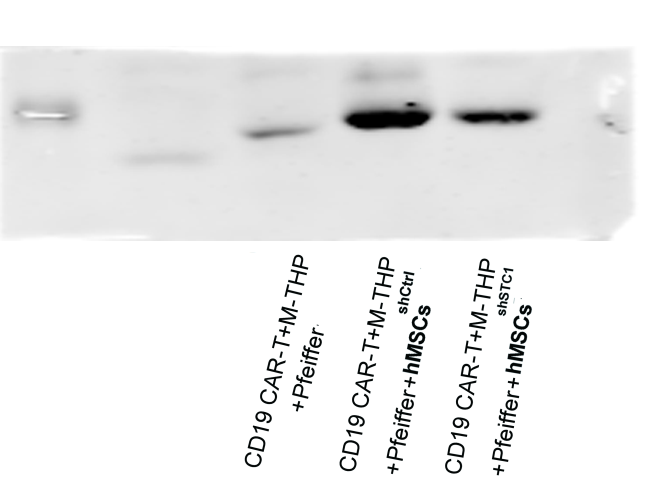

Supplement: Figure 2—source data 4. [file elife-82934-fig2-data4.zip › Fig2F IDO.tif]

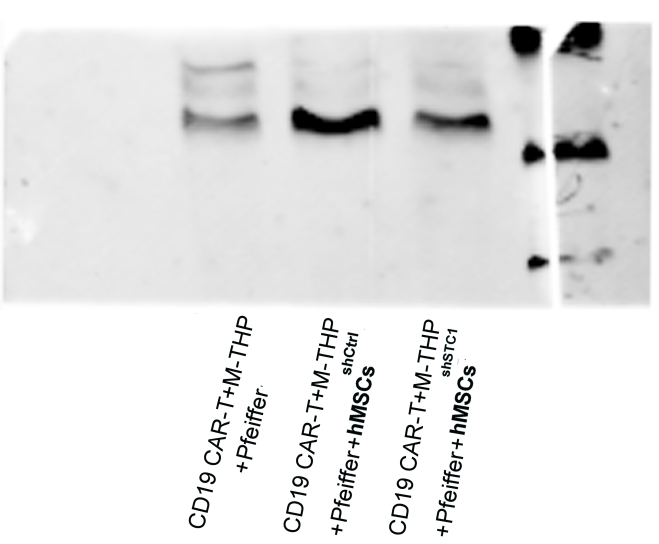

Supplement: Figure 2—source data 4. [file elife-82934-fig2-data4.zip › Fig2F PD-L1 repeat.tif]

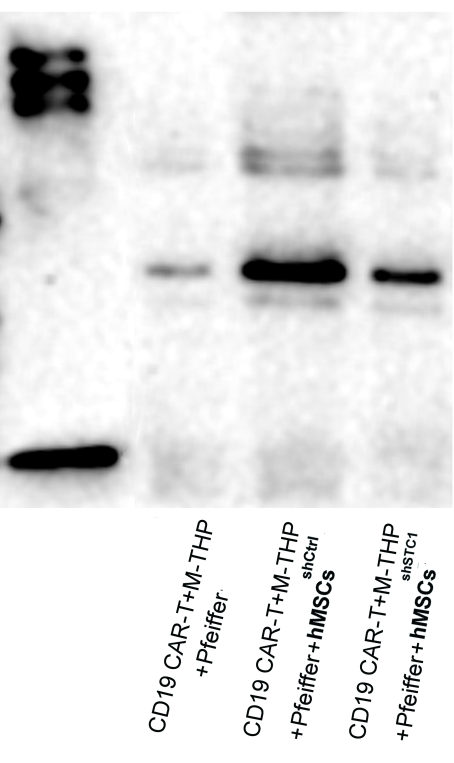

Supplement: Figure 2—source data 4. [file elife-82934-fig2-data4.zip › Fig2F PD-L1.tif]

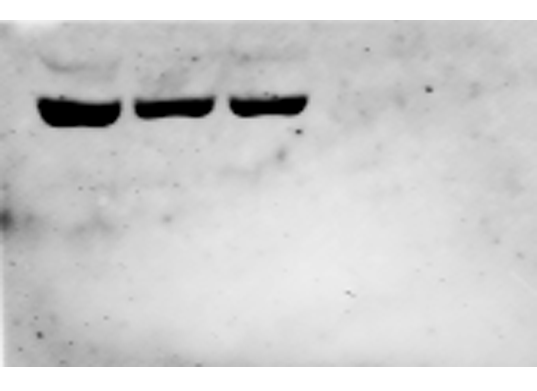

Supplement: Figure 2—source data 5. [file elife-82934-fig2-data5.zip › Fig2F GAPDH repeat.tif]

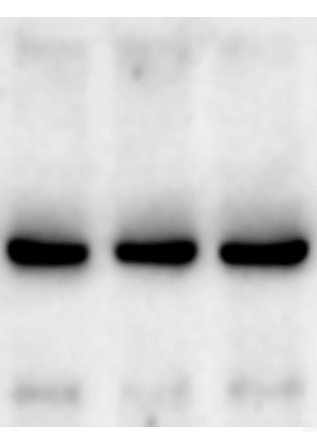

Supplement: Figure 2—source data 5. [file elife-82934-fig2-data5.zip › Fig2F GAPDH.tif]

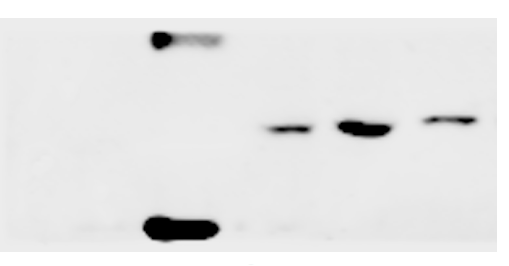

Supplement: Figure 2—source data 5. [file elife-82934-fig2-data5.zip › Fig2F IDO repeat.tif]

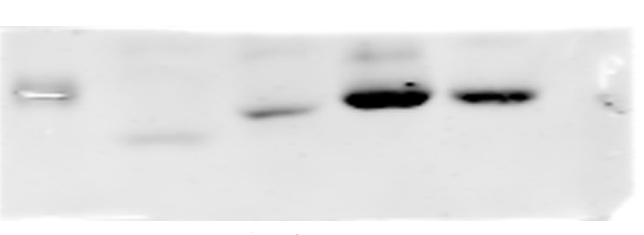

Supplement: Figure 2—source data 5. [file elife-82934-fig2-data5.zip › Fig2F IDO.tif]

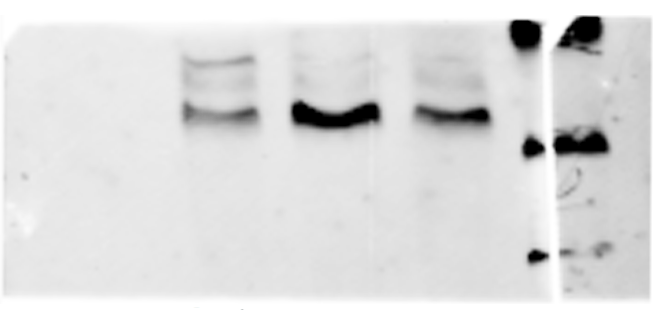

Supplement: Figure 2—source data 5. [file elife-82934-fig2-data5.zip › Fig2F PD-L1 repeat.tif]

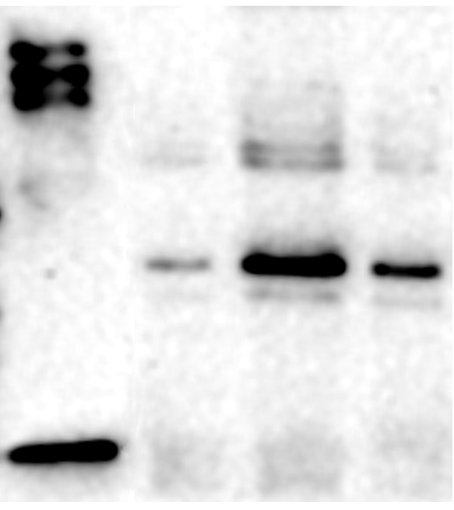

Supplement: Figure 2—source data 5. [file elife-82934-fig2-data5.zip › Fig2F PD-L1.tif]

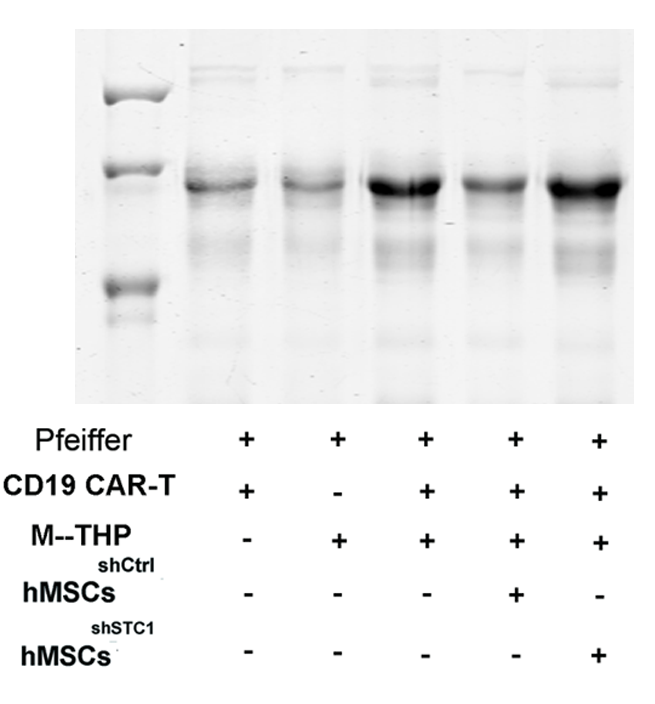

Supplement: Figure 3—source data 1. [file elife-82934-fig3-data1.zip › Fig3A AIM2 repeat.tif]

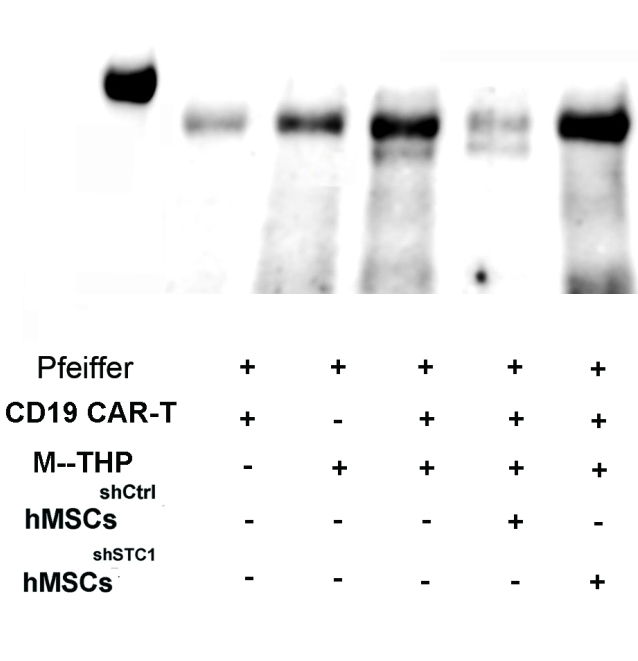

Supplement: Figure 3—source data 1. [file elife-82934-fig3-data1.zip › Fig3A AIM2.jpg]

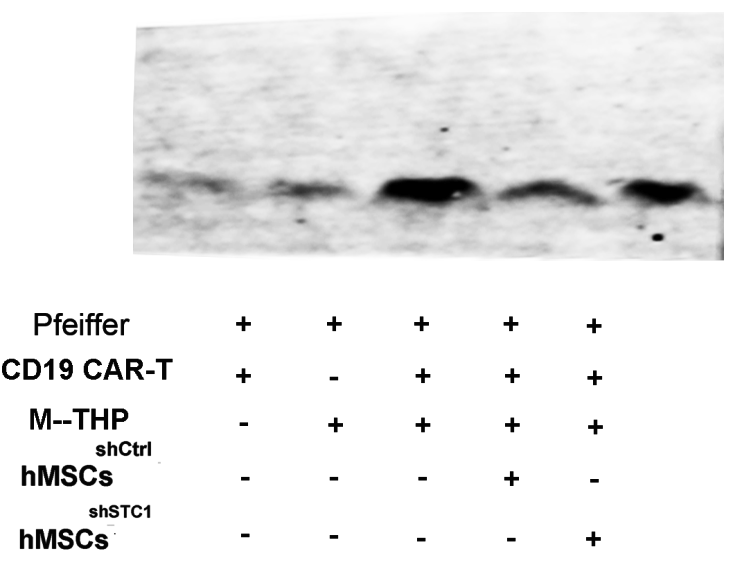

Supplement: Figure 3—source data 1. [file elife-82934-fig3-data1.zip › Fig3A caspase-1 p20 repeat.tif]

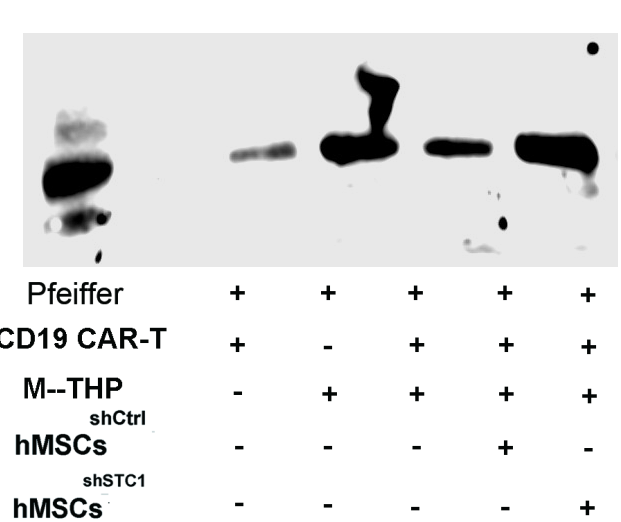

Supplement: Figure 3—source data 1. [file elife-82934-fig3-data1.zip › Fig3A caspase-1 p20.tif]

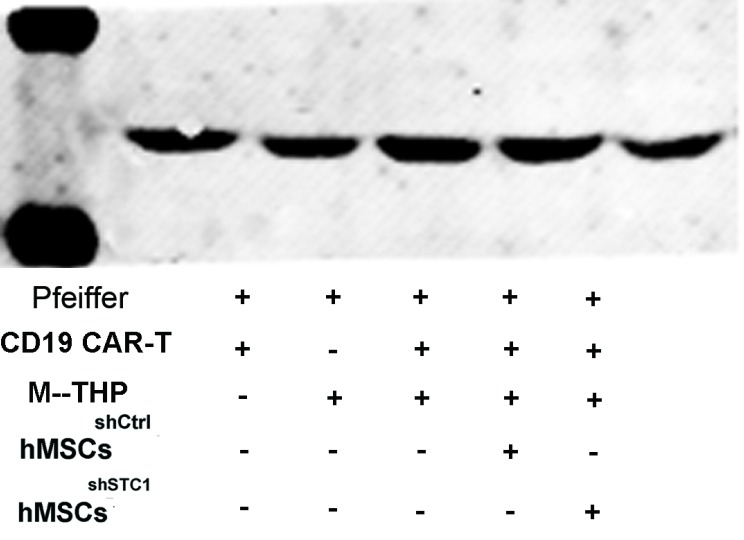

Supplement: Figure 3—source data 1. [file elife-82934-fig3-data1.zip › Fig3A GAPDH .tif]

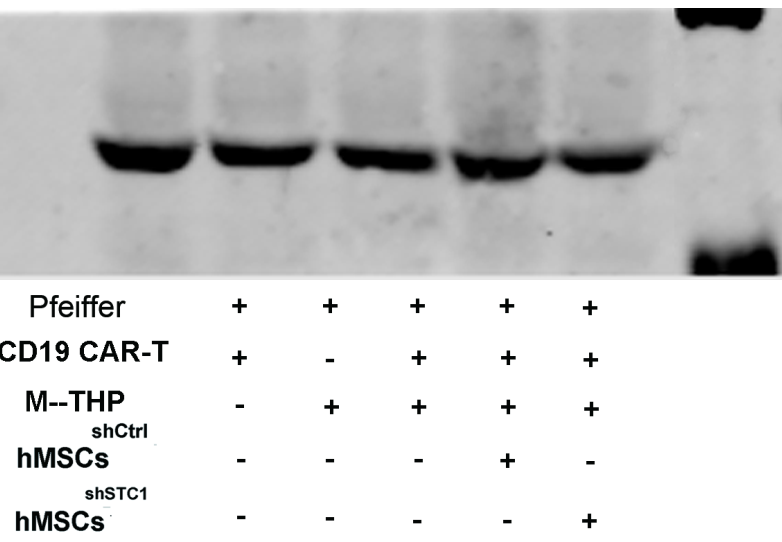

Supplement: Figure 3—source data 1. [file elife-82934-fig3-data1.zip › Fig3A GAPDH repeat.tif]

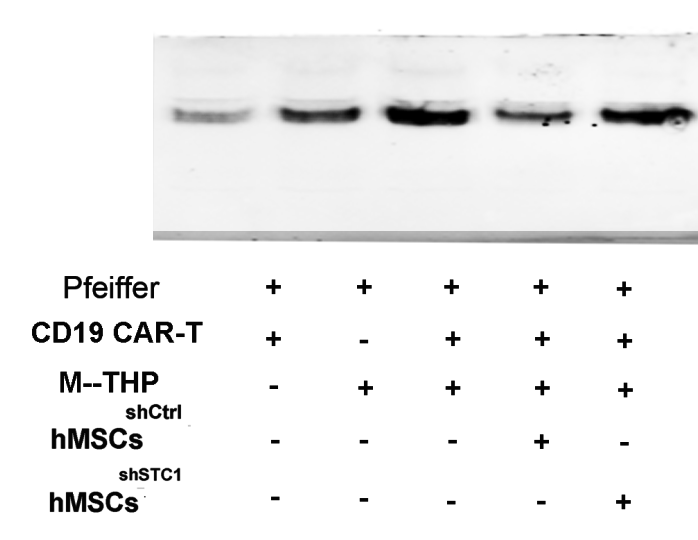

Supplement: Figure 3—source data 1. [file elife-82934-fig3-data1.zip › Fig3A IL-1 beta repeat.tif]

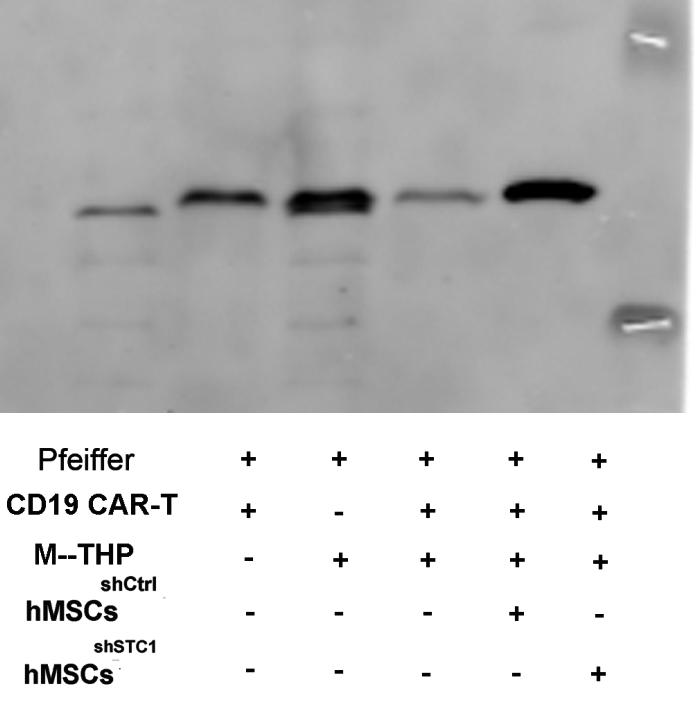

Supplement: Figure 3—source data 1. [file elife-82934-fig3-data1.zip › Fig3A IL-1 beta.tif]

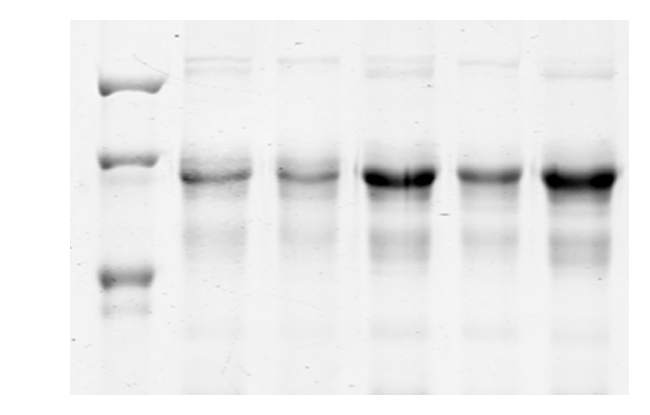

Supplement: Figure 3—source data 2. [file elife-82934-fig3-data2.zip › Fig3A AIM2 repeat.tif]

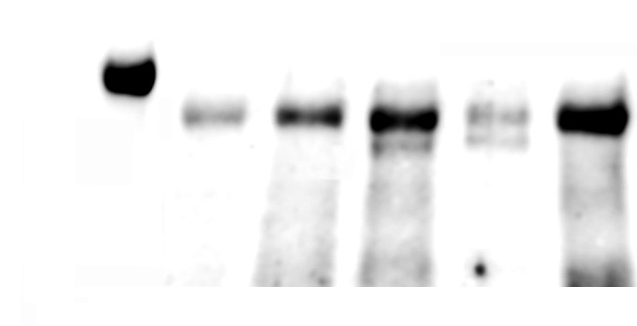

Supplement: Figure 3—source data 2. [file elife-82934-fig3-data2.zip › Fig3A AIM2.jpg]

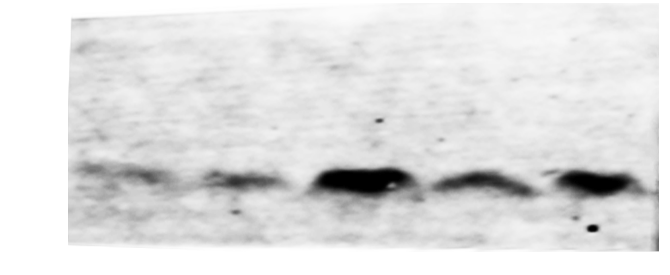

Supplement: Figure 3—source data 2. [file elife-82934-fig3-data2.zip › Fig3A caspase-1 p20 repeat.tif]

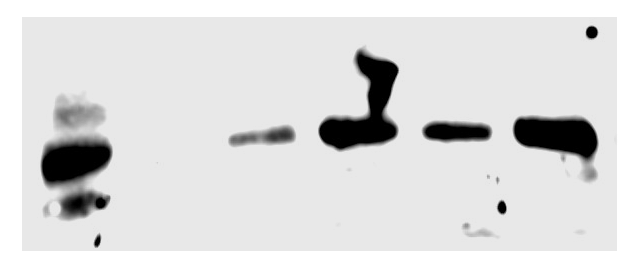

Supplement: Figure 3—source data 2. [file elife-82934-fig3-data2.zip › Fig3A caspase-1 p20.tif]

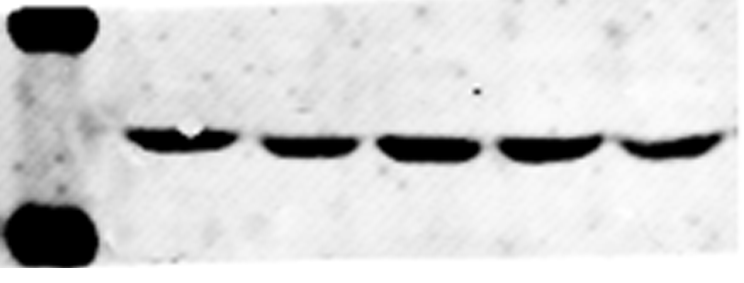

Supplement: Figure 3—source data 2. [file elife-82934-fig3-data2.zip › Fig3A GAPDH .tif]

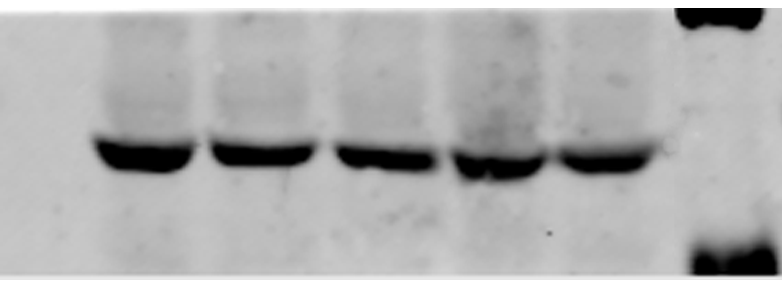

Supplement: Figure 3—source data 2. [file elife-82934-fig3-data2.zip › Fig3A GAPDH repeat.tif]

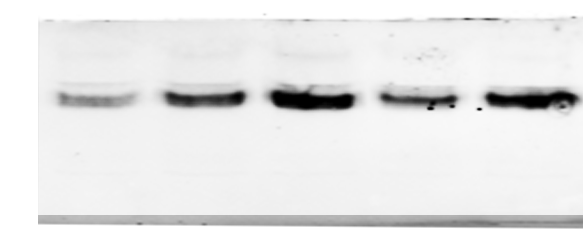

Supplement: Figure 3—source data 2. [file elife-82934-fig3-data2.zip › Fig3A IL-1 beta repeat.tif]

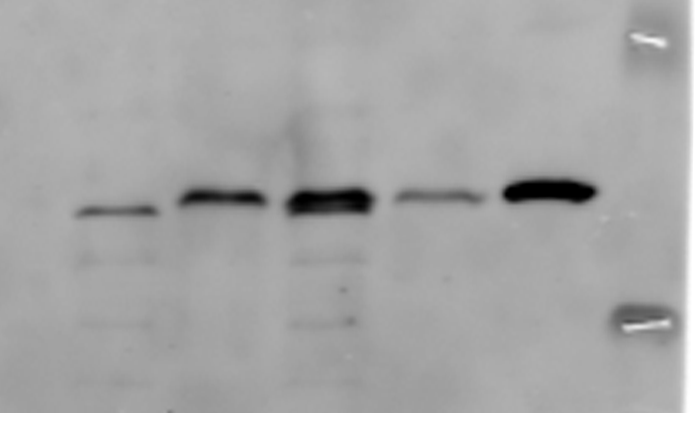

Supplement: Figure 3—source data 2. [file elife-82934-fig3-data2.zip › Fig3A IL-1 beta.tif]
